# Tissue-in-a-Tube: three-dimensional *in vitro* tissue constructs with integrated multimodal environmental stimulation

**DOI:** 10.1016/j.mtbio.2020.100070

**Published:** 2020-07-28

**Authors:** A. Shahin-Shamsabadi, P.R. Selvaganapathy

**Affiliations:** a School of Biomedical Engineering, McMaster University, Canada; b Department of Mechanical Engineering, McMaster University, Canada

**Keywords:** 3D *in vitro* model, Dynamic microenvironment, Perfusion, Mechanical/electrical stimulation, Multiculture system, Cell patterning

## Abstract

Three-dimensional (3D) *in vitro* tissue models are superior to two-dimensional (2D) cell cultures in replicating natural physiological/pathological conditions by recreating the cellular and cell-matrix interactions more faithfully. Nevertheless, current 3D models lack either the rich multicellular environment or fail to provide appropriate biophysical stimuli both of which are required to properly recapitulate the dynamic *in vivo* microenvironment of tissues and organs. Here, we describe the rapid construction of multicellular, tubular tissue constructs termed Tissue-in-a-Tube using self-assembly process in tubular molds with the ability to incorporate a variety of biophysical stimuli such as electrical field, mechanical deformation, and shear force of the fluid flow. Unlike other approaches, this method is simple, requires only oxygen permeable silicone tubing that molds the tissue construct and thin stainless-steel pins inserted in it to anchor the construct and could be used to provide electrical and mechanical stimuli, simultaneously. The annular region between the tissue construct and the tubing is used for perfusion. Highly stable, macroscale, and robust constructs anchored to the pins form as a result of self-assembly of the extracellular matrix (ECM) and cells in the bioink that is filled into the tubing. We demonstrate patterning of grafts containing cell types in the constructs in axial and radial modes with clear interface and continuity between the layers. Different environmental factors affecting cell behavior such as compactness of the structure and size of the constructs can be controlled through parameters such as initial cell density, ECM content, tubing size, as well as the distance between anchor pins. Using connectors, network of tubing can be assembled to create complex macrostructured tissues (centimeters length) such as fibers that are bifurcated or columns with different axial thicknesses which can then be used as building blocks for biomimetic constructs or tissue regeneration. The method is versatile and compatible with various cell types including endothelial, epithelial, skeletal muscle cells, osteoblast cells, and neuronal cells. As an example, long mature skeletal muscle and neuronal fibers as well as bone constructs were fabricated with cellular alignment dictated by the applied electrical field. The versatility, speed, and low cost of this method is suited for widespread application in tissue engineering and regenerative medicine.

## Graphical abstract

**Figure:**
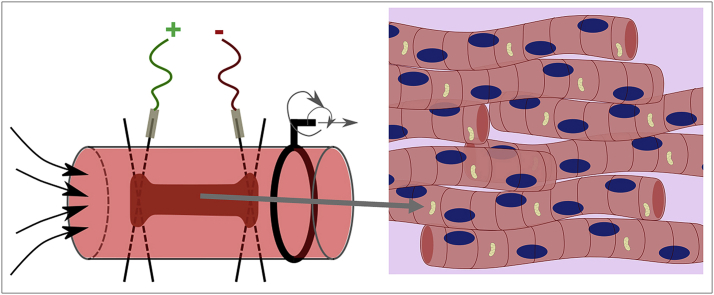

## 1 Introduction

Improved *in vitro* models for human tissues and organs are sought for drug discovery and understanding disease mechanisms as they simulate the *in vivo* conditions better than existing two-dimensional (2D) cell culture systems and can also mimic human physiology better as compared with animal models. Several approaches have been investigated to address these limitations such as organ-on-a-chip devices that recreate tissue and organ interfaces *in vitro* [1,2] with precise structural, mechanical, electrical, and fluidic control over customized cellular environments [3]. Alternatively, three-dimensional (3D) models that recreate the complex cell-cell and cell-matrix interactions and incorporate transport-induced features such as natural gradient of gases, nutrients, and signaling factors have been developed as well in the form of multicellular spheroids [4] and using bioprinting techniques [5].

Organ-on-a-chip systems incorporate the major components of physiological systems that were previously missing from 2D *in vitro* models such as the multicellular patterning and interfaces of organs, presence of flow, and electrical and/or mechanical stimulation [1]. This interface was traditionally created by incorporating plastic porous membranes into microfluidic channels [1] which prevented direct physical contact between cell types. Recognizing this limitation, later versions have been created with much thinner membranes. However, fabricating such devices and integrating thin and fragile membranes to the microfabricated chips requires special expertise and the methods that are expensive and time-consuming. Furthermore, traditional organ-on-a-chip systems were capable of recreating a dynamic microenvironment but were essentially 2D in nature which was non-physiological. Later versions were developed to overcome this limitation by incorporating microtissues, cell-laden hydrogels, multicell layers, and living tissue biopsies to make them physiologically relevant, albeit with increased complexity in fabrication [1].

Multicellular spheroid models preserve the interactions between cells and their matrices that are found *in vivo* and attempt to recapitulate gradients in nutrients and signaling molecules that have a strong influence on cellular behavior resulting in gene and protein expression profiles that are closer to *in vivo* conditions [6]. Multicellular spheroids can be formed by incorporation of extracellular matrices (ECMs) to embed the cells in the initial construct [4,[7], [8], [9]] or by matrix-free methods that rely on formation of initial loose cell aggregates that become more compact by subsequent secretion of extracellular matrices by the cells [10]. Matrix-free methods are dependent on the cell type used to efficiently self-assemble and the initial conditions as well as settling of the cells can have an effect on the 3D shapes formed. Furthermore, as they need to produce their own ECM, the assembly process takes a long time (several days), and the matrix composition can be variable [[11], [12], [13]]. Matrix-based techniques overcome some of these limitations by using a well-defined ECM material that not only creates a controlled environment [14] but also aids in the initial self-assembly process and makes assembly less prone to settling [4,7]. Despite these advantages, many of the current techniques are limited in the cell density, fabrication speed, control over positioning of different cell types, and creation of tissue/organ interfaces. More importantly, formation of necrotic cores and the inability to grow them beyond a certain size because of mass transport limitations is one of the key limitations of multicellular spheroid models, especially for applications other than modeling avascular stage of the cancerous tissues. Finally, it is also difficult to incorporate biophysical cues such as electrical and mechanical stimulation that is increasingly being considered important to recreate the *in vivo* environments, because of their form factor.

For this reason, non-spherical 3D constructs have been fabricated to not only avoid formation of necrotic cores but also to introduce biophysical signals (electrical and mechanical, stretch or shear) either during the formation or the testing phase. Most of these techniques form small (millimeter size) constructs, often made of one cell type, with long assembly time and are capable of introducing one or at most two biophysical signals. For example, 3D constructs solely composed of cardiomyocytes have been converted to functional cardiac tissue using pneumatic methods to apply a periodic stretch over a period of 5 days [15] without the ability to provide other biophysical stimulus. 3D skeletal muscle constructs of a few millimeters in length have also been fabricated using self-assembly around anchoring pins but without the ability to introduce different types of cells and ECMs and control over their precise positioning [[16], [17], [18]]. In addition, the fabrication process takes a few days, and the use of rigid pins [16,17] restricts the application of external mechanical deformation, whereas the compliant pins [18] were used to measure the self-generated traction force and not provide stimulus. Ability to precisely position cells to form neuromuscular interfaces has been demonstrated using a device that required a microfabrication process and a lengthy cell culture period [19,20]. Annular cardiac tissue constructs of a few millimeters in length have also been fabricated by self-assembly of collagenous bioink around suture wires [21] or hollow fibers [22] over 7 days. External electrodes were then incorporated to impart electrical stimulation to these constructs. A variation of the method using compliant suture wires as anchors was also demonstrated to sense the contractile tension in the tissue construct [23] but with no means to impart external stretch or electrical signals.

In some examples, multiple biophysical stimuli have been combined albeit with simple and small (<1 cm) 3D constructs with single-cell type and no ability to pattern cells. An interesting example is a device that enabled simultaneous mechanical (static stretch) and electrical stimulation of a 3D construct composed of collagen-embedded cardiomyocytes [24]. It took 3 days to form the self-assembled construct of 7 mm in length, and a complicated pneumatic system was used to impose a static deformation on a microfabricated mold without any perfusion control. Alternatively, perfusion bioreactors where cells seeded in 3D scaffolds (without any control over their positioning) and incorporating electrodes for stimulation [25,26] have been used to combine perfusion with electrical stimulation.

In summary, existing methods do not combine all the required features including rapid self-assembly of 3D tissue constructs, ability to precisely position different cell types and pattern them, ability to scale sizes of the constructs, and the ability to incorporate all the three important biophysical stimuli, stretch, shear, and electric. More importantly, they also involve customized molds, tools, and equipment that make it difficult to implement without appropriate engineering expertise. In this article, we develop a method to overcome these limitations. Here, large constructs with cylindrical shape and uniform and well-defined mass transport properties without necrotic cores are created with high cell density, with multiple cell types positioned in predefined patterns and with clear interfaces, combined with multitude of electrical/mechanical stimulation to create a dynamic environment. The cylindrical format enables scalability and construction of various sizes (mm to cm). The format inherently is suited for perfusion of media to support metabolic needs of cells and create biomimetic shear conditions resulting in a physiologically relevant model that very closely mimics the *in vivo* conditions. This technique is simple (no microfabrication steps required) and fast (only a few hours culture time before stable tissue constructs are formed) without the need for specific fabrication equipment, has the ability to control positioning of multiple cell types/ECM materials with unidirectional or multidirectional crosstalk between them. It can also be used to fabricate macrostructures with different shapes that can be used as cell vehicles for implantation or as *in vitro* models for applications such as drug screening.

## 2 Materials and methods

**Fig. 1:**
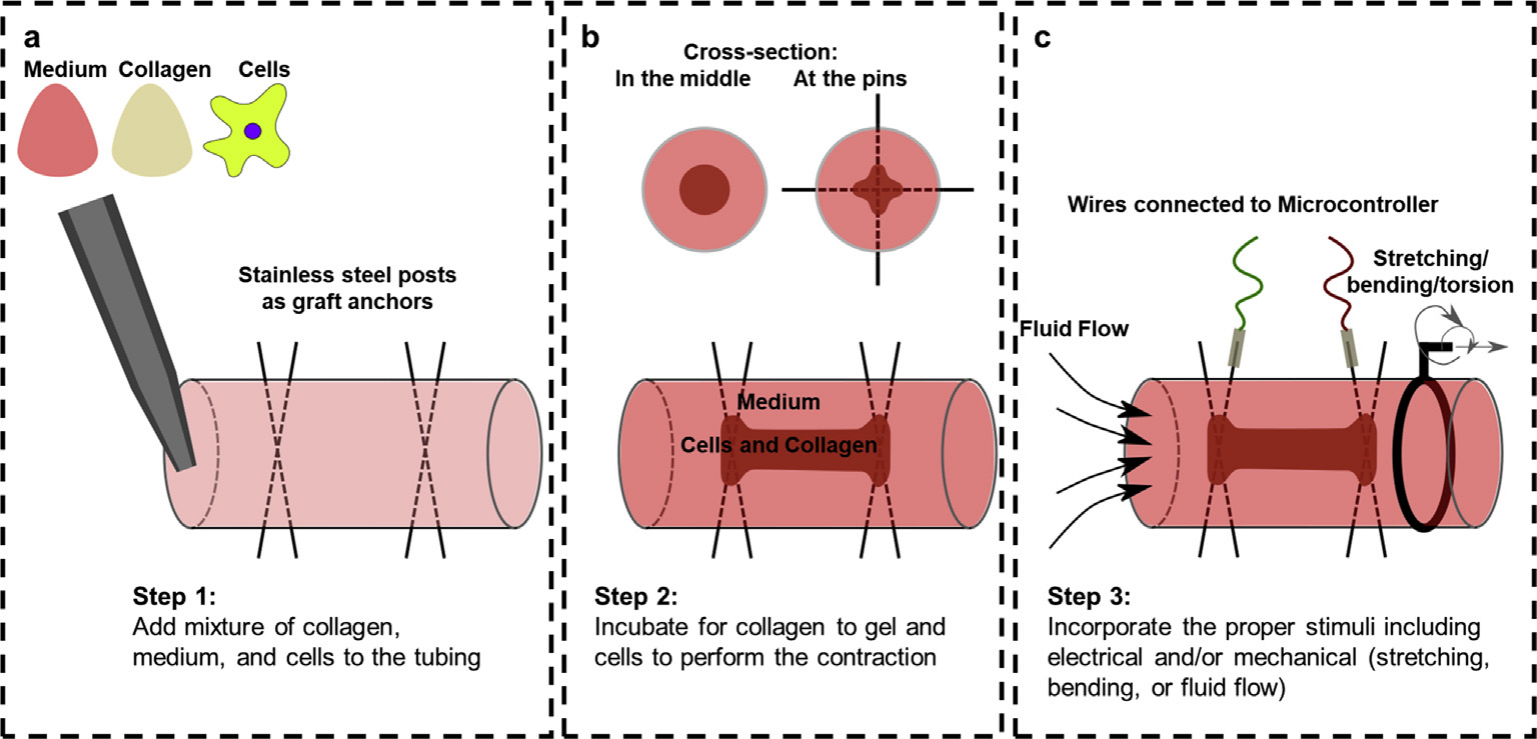
Schematic of the process; **a)** Tubing is filled with neutralized collagen, medium, and cell solution; **b)** After collagen gels and cells adhere to it, collagenous construct is formed within the tubing by clinging to the stainless-steel pins as support; **c)** Fluid flow, electrical stimulation, and deformation of tubing (stretching, bending, and torsion) can be applied to create a 3D dynamic environment for cells. Different volume ratios of collagen to medium, cell densities, and tubing sizes can be used to change the compactness and size of the construct.

Collagenous constructs of cells inside a silicone tubing and anchored to the metallic pins were formed using a process of self-assembly that has been used previously for spheroidal and non-spheroidal structures [27]. There, a mixture of cells, medium, and neutralized bovine collagen type I were added to a polydimethylsiloxane (PDMS) mold with the specific form that defined the final shape of the construct. A stable construct forms after rapid gelation of collagen and attachment of cells to it that causes the consolidation of the construct. In the current technique, replacing PDMS molds with silicone tubing enables production of solid tube-like constructs which are anchored on the stainless-steel pins (Fig. 1). The silicone tubing is widely available, do not require any special fabrication process, and can be connected using connectors to form complex networks. It is also gas permeable. The pins can be inserted into the tubing without leakage and serve as anchoring points axially to shape the construct formation and to apply axial tension as the construct forms.

Once the self-assembly process is complete, an annular gap forms between the construct and the tubing that can be used for perfusion purposes and to apply shear forces on the construct. The inserted pins can be connected to a microcontroller to apply electrical stimulation to the construct, and the tubing itself can be stretched, bent, or torqued to create different types of mechanical deformation in the construct. This setup allows formation of collagenous constructs in a very short process (4–6 h) that can be maintained and monitored in a true 3D and dynamic environment with different types of stimuli.

### 2.1 Cell culture

Different types of cells were used in the current study for different purposes. Michigan Cancer Foundation-7 (MCF-7) breast cancer cells were cultured in Dulbecco's Modified Eagle Medium (DMEM) (with l-glutamine and high glucose, Gibco), supplemented with 10 %V/V fetal bovine serum (FBS) (Canada origin, ThermoFisher) and 1%V/V penicillin-streptomycin (10,000 U/mL, ThermoFisher) until 70% confluent. C2C12 myoblast cells were grown in the same DMEM, supplemented with 10%V/V heat-inactivated FBS (HI-FBS) (Canadian origin) and 1%V/V penicillin-streptomycin. For differentiation purposes, these cells were cultured in DMEM supplemented with 2%V/V of horse serum (ThermoFisher) and 1%V/V penicillin-streptomycin and 0.1% insulin (Insulin-Transferrin-Selenium, 100X, ThermoFisher, Catalogue number 41400045). SH-SY5Y neuroblastoma cells were cultured in DMEM/F-12 (ThermoFisher, with l-glutamine) medium supplemented with 10% HI-FBS and 1% penicillin-streptomycin. For differentiation of these cells, DMEM/F12 was supplemented with 1% heat-inactivated FBS, 1% N2 supplement, and 1 μM retinoic acid. Red fluorescent protein–tagged human umbilical vein endothelial cells (HUVECs) were grown in EBM-2 medium. Osteoblast-like cells from Saos-2 osteosarcoma cell line were cultured in McCoy's medium (ThermoFisher, with l-glutamine) supplemented with 15% FBS and 1% penicillin-streptomycin.

### 2.2 Tissue-in-a-tube: fabrication and optimization

MCF-7 cells were used for characterization purposes to study effect of collagen to medium ratio (CMR), cell density, tubing size, and distance between the stainless-steel pins. 1:1, 1:3, and 1:5 ratios were used, while other parameters were kept constant at 2 × 10^6^ cells/mL, tubing with 3 mm inner diameter (ID) and pins being 2 cm apart. The 1:1, 1:3, and 1:5 ratios corresponded to ~2,5 mg/mL, 1,25 mg/mL, and 0,8 mg/mL of effective collagen concentration in the final solution. Effect of cell density was studied by using bioinks containing 1, 2, and 3 × 10^6^ cells/mL of the bioink with 1:3 CMR and 2 cm-wide pins in 3 mm ID tubing. Effect of tubing size was studied by using tubing with 1, 3, and 7 mm ID, termed as thin, medium, and thick, respectively, while 1:3 CMR, 2 × 10^6^ cells/mL bioink, and 2 cm wide pins were used. To study the ability to form constructs with different lengths, tubing with 3 mm ID was used with a bioink with 1:3 CMR and 2 × 10^6^ cells/mL density, but pins were kept 2 and 4 cm apart. After filling the tubing with the bioink in each case, incubation at 37°C was performed for 4 more hours until shrinkage of the stable constructs was done. Images of the samples were taken using a dissecting microscope (Infinity Optical Systems). Bioink was prepared by dispersing cells in the required volume of the medium and then addition of neutralized bovine collagen I (ThermoFisher, 5 mg/mL). Collagen was neutralized by addition of 0.1 M sodium hydroxide in DI water. Stainless steel 304 wire (McMASTER-CARR) with 0.5 mm diameter were used as pins, and at each point, two pins perpendicular to each other were inserted in the tubing to provide proper anchorage for the constructs.

Live/dead staining was performed using the kit (ThermoFisher) following the provided protocol. Briefly, calcein-AM and ethidium homodimer-1 were diluted in the medium and added to the samples (formed with 1:3 CMR and 2 × 10^6^ cells/mL density in tubing with medium thickness and with 2 cm apart pins) 4 h after process was started followed by 1 h of incubation. Images of the samples were taken using an inverted fluorescent microscope with 4X magnification and proper filters.

### 2.3 Controlled cellular interfaces

Formation of clear and continuous interface between regions containing different cell types in a contiguous tissue construct was shown in both axial and radial configurations. MCF-7 cells were stained with either green DiO or red DiI fluorescent cell trackers (ThermoFisher). For the axial configuration, half of the tubing was filled with the bioink containing green stained cells (1:3 CMR, 2 × 10^6^ cells/mL solution). After half hour incubation when the collagen had gelled but the cells had not attached to the ECM to apply significant traction forces, the other half of the tubing was filled with the same bioink but with red stained cells. For radial configuration, the whole tubing was filled with green stained cells' bioink (1:3 CMR, 2 × 10^6^ cells/mL solution). After 2 h of incubation that shrinkage was performed, extra medium was extracted, and a 1:3 CMR bioink with 1 × 10^6^ cells/mL was added followed by further incubation. Fluorescent images were taken before and after addition of each bioink using a ChemiDoc™ MP imaging system (Bio-Rad).

### 2.4 Complex macrostructures

Macrostructures with different patterns including bifurcated patterns and columns with varying axial thicknesses were formed using HUVECs. For bifurcated patterns, three 3 mm ID tubing, each 2 cm in length were connected to each other using a Y-shaped connector. At the end of each tubing, two perpendicular pins were inserted as anchor pins, and the entire connection was filled with 1:3 CMR and 2 × 10^6^ cells/mL solution of HUVECs. After 1 h of incubation that collagen had gelled and some shrinkage was observed, pins were removed, and bifurcated macrostructure was retrieved from the tubing. Columns with descending thicknesses were formed by connecting 2 cm long tubing with 7, 3, and 1 mm IDs using proper connectors, respectively. Perpendicular pins were inserted in the middle of each tubing, and the same bioink as before was added. Macrostructure was retrieved after 1 h of incubation. Fluorescent images were taken using the same ChemiDoc™ MP imaging system, before and after samples were taken out of the connected tubing.

### 2.5 Dynamic environment

C2C12 constructs were formed with 1:3 CMR and 2 × 10^6^ cells/mL bioink in tubing with 3 mm ID and 2 cm apart pins in the cells' growth medium. After 24 h, the medium was switched to the cells’ differentiation medium, and at the same time, a step electrical signal with peak to peak voltage of 10 V (5 V/cm) and frequency of 50 Hz was applied (5 samples in parallel). An open source microcontroller, Arduino Uno R3, was used to create this signal and to control a motor that was used for perfusion (flow rate of 0.1 mL/min for 1 min every 12 h). The code used for programming the microcontroller that controls the bioreactor is included in Supplementary Box 1. This group of samples were named ‘Dynamic’. Samples were kept in this condition for 3 more days. As control groups, samples formed in the tubing for 24 h but retrieved from it and kept in 6 well plates in 2 mL differentiation medium (‘In Well’ group), and samples kept in tubing with differentiation medium but without electrical stimulation (‘In Tube’ group) were considered. At day 4, samples were taken out of the tubing, and images were taken using the dissecting microscope used previously. ImageJ software was used to measure thickness of the constructs before and after releasing them from the anchor pins and were compared to the ‘In Well’ samples.

Three samples for each condition (n = 4) were digested using a 0.5 mL of 2 V/V% collagenase/dispase (Sigma-Aldrich) solution in PBS (stock solution was 100 mg/mL collagenase/dispase in DI water). After digestion was done, another 0.5 mL of 0.5% Triton X-100 in PBS was added to lyse the samples. Pierce BCA (ThermoFisher) kit was used to measure the protein content of each sample by using two 25 μL aliquots of lysate solution in 96-well plates, where 200 μL of kit solution (50:1 ratio mixture of parts A and B of the kit) was added to each well. Absorbance was measured at 562 nm after 30 min incubation at 37°C in duplicate reading for each sample. Mixture of collagenase/dispase and Triton X-100 solutions was used as control, and its value was subtracted from the samples.

Three more samples for each condition were fixed in 2% formaldehyde solution in DI water for 1 h. After fixation was done, samples were washed with warm PBS two times and 1 mL of PBS containing 25 μL of Alexa Fluor™ 488 Phalloidin (ThermoFisher) stock solution (300 units dissolved in 1.5 mL methanol), and 0.2% Tween-20 as permeabilizing agent was added with 1 h incubation at room temperature. After washing with PBS, samples were counterstained with 1 mL PBS containing 1 μL of DAPI (4′,6-diamidino-2-phenylindole, dihydrochloride, ThermoFisher) stock solution (10 mg/mL in DI water) for 30 min. Imaging was performed using an inverted fluorescent microscope (Olympus, USA) with DAPI and FITC filters with Ex/Em of 381–392/417–477 and 475–495/512-536, respectively. Live/dead staining and imaging was performed as before on the ‘Dynamic’ and ‘In Tube’ groups after samples were taken out of the tubing at day 4.

Constructs were also formed with SH-SY5Y and Saos-2 cells in 3 mm ID tubing with 1:3 CMR and 2 cm apart pins, while cell density was 4 × 10^6^ cells/mL for SH-SY5Y cells and 2 × 10^6^ cells/mL for Saos-2 cells. Differentiation of SH-SY5Y was started at day 1 by switching to their differentiation medium, and in both cases, electrical stimulation was started after 1 day and continued for 5 days with 10 V peak to peak and 50 Hz frequency.

### 2.6 Statistical analysis

Data are reported as mean ± standard deviation, and statistical analysis was performed using one-way analysis of variance test in GraphPad Prism with an accepted statistical significance (*p*-value) less than 0.05. Significant outlier data points were detected using Grubbs’ test.

## 3 Results and discussion

### 3.1 Tissue-in-a-tube: fabrication and optimization

A new biofabrication approach termed as Tissue-in-a-Tube has been developed to form highly dense multicellular cylindrical constructs, rapidly with the ability to incorporate electrical and/or mechanical stimuli to cells in a 3D environment along with continuous medium perfusion (Fig. 1). Silicone tubing with stainless steel pins inserted in it were used as the molds for the assembly of 3D collagenous constructs. These pins, inserted at specific locations, act as anchors and direct the self-assembly of the constructs between them when appropriate bioinks are injected into the silicone tubing (Fig. 1a). They also allow application of electric field axially over the construct during or after its formation process. The self-assembly process leads to shrinkage of the collagenous bioink into a dense construct at the center of the tubing leaving a uniform concentric gap around it that can be used for perfusion of nutrients, removal of waste, and to apply shear stimulus (Fig. 1b). The silicone tubing is also gas permeable and thus allows gas exchange to support long-term tissue culture. It is also flexible, and mechanical deformation of it such as stretching, bending, or torsion can induce similar effect on the anchored tissue constructs (Fig. 1c). Single or multiple stimuli can be applied to the constructs in a time-dependent manner depending on the cell types used in the biofabrication process. The technique is simple, low-cost, and rapid. It can be used with a variety of cell types including epithelial (such as MCF-7 breast cancer cells) and endothelial (such as HUVECs) cells, skeletal muscle cells (such as C2C12 cells), neuronal cells (such as SH-S5Y5 cells), and bone cells (such as Saos-2 cells), either alone or in co-culture as shown in detail in the following sections. It can also form larger constructs with multitude of stimuli applied simultaneously.

**Fig. 2:**
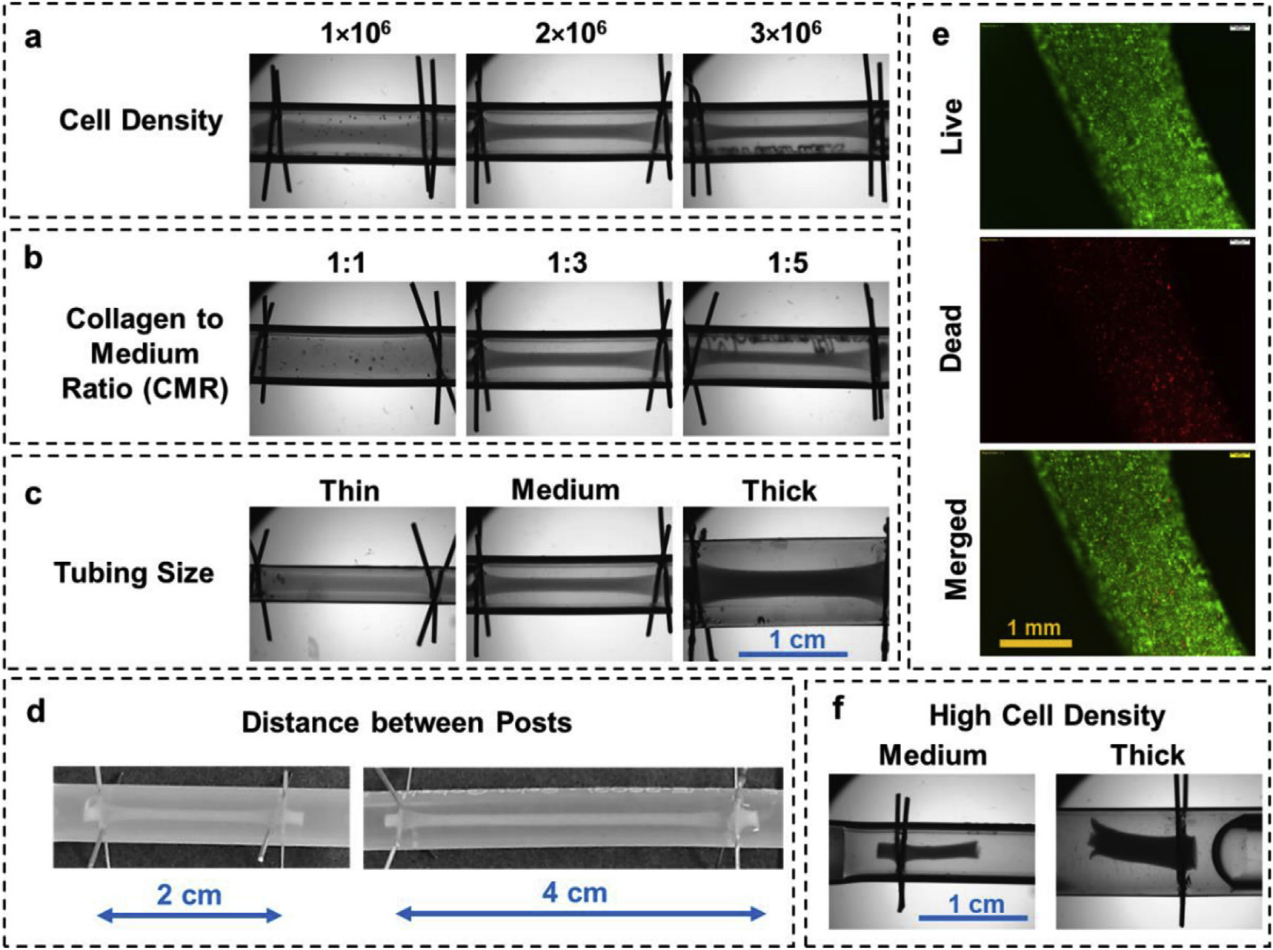
Characterization of parameters effective on the Tissue-in-a-Tube process using MCF-7 cells; Effect of **a)** cell density (with 1:3 CMR and medium thickness tubing); **b)** CMR (with density of 2 × 10^6^ cells/mL and medium thickness tubing); **c)** Tubing thickness (with 1:3 CMR and density of 2 × 10^6^ cells/mL). **d)** Effect of distance between anchor pins, longer constructs can be formed by increasing the length of the tubing (with 1:3 CMR and density of 1 × 10^6^ cells/mL); **e)** Live/dead stained samples 4 h after process was started. **f)** Increasing the cell density would increase the contraction which leads into developing a tear in the structure. All images are taken 4 h after assembly. In each case, n = 3 was used to ensure the process is repeatable.

Different parameters such as cell density, initial collagen to medium ratio (CMR), and tubing size (ID) can affect the dimensions of the formed construct as well as its compactness which was characterized using MCF-7 cells with the epithelial phenotype characteristic. Increasing the cell density and CMR, or decreasing the tubing ID, decreased the thickness of the construct (Fig. 2a–c). For instance, increasing the cell density from 1 to 2 and 3 × 10^6^ cells/mL while the CMR and tubing ID were kept at 1:3 and 3 mm decreased the diameter of the tubular graft from 1854 ± 45 to 1375 ± 41 and 1028 ± 51 μm, respectively (n = 4). It should be noted that the formation of the construct and the contraction leads to a dramatic increase in its cell density (final densities of ~0.28 × 10^7^, ~1.06 × 10^7^, and ~2.54 × 10^7^ cells/mL for initial densities of 1, 2, and 3 × 10^6^ cells/mL, respectively, when CMR and tubing ID of 1:3 and 3 mm were used). Starting with a higher cell density will also result in a higher relative increase in the final construct as more shrinkage happens because of higher cell-cell interactions. For example, the total increase in the final density of constructs with seeding densities of 1, 2, and 3 × 10^6^ cells/mL was 2.8, 5.3, and 8.5, respectively.

Increase in cell density leads to increase in cell-cell and cell-ECM interactions that facilitate higher traction forces and increased consolidation of the construct [27]. Longer constructs were formed by increasing the distance between the anchor pins while maintaining the diameter of the tubular structure (Fig. 2d). The rate of contraction because of self-assembly is dramatic in the first 4 h and decreases over the next 20 h after which the size of the construct stabilizes (Supplementary Fig. 1).

Bioinks with CMR of 1:3 and cell density of 2 × 10^6^ cells/mL seeded into tubing with anchor pin spacing of 2 cm and 4 cm produced correspondingly long constructs with minimal change in their diameter (1375 ± 41 vs. 1320 ± 89 μm for 2 and 4 cm apart pins, respectively). Since construct formation process is fast (~4 h), the cells were viable, and only a small number of dead cells can be observed with uniform distribution rather than formation of necrotic regions (Fig. 2e). Interestingly, the number of dead cells close to the anchoring pins was slightly higher than in the rest of the construct which could be because of higher traction forces in those regions (Supplementary Fig. 2). Increasing the cell density increased the amount of internal strain generated in the construct, resulting in excessive contraction which led to its catastrophic failure (Fig. 2f).

### 3.2 Controlled cellular interfaces/complex macrostructures

**Fig. 3:**
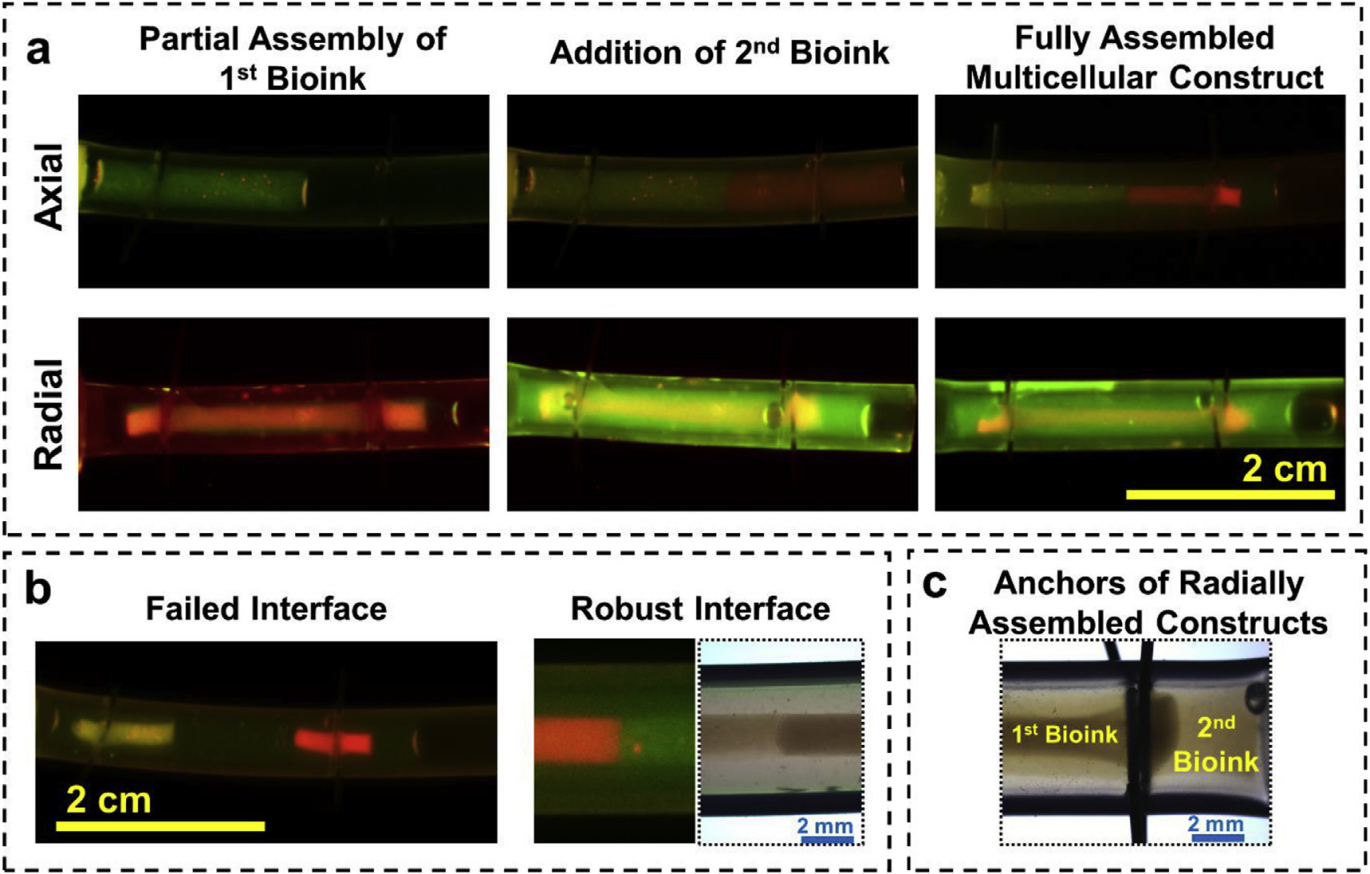
**a)** Controlled graft interfaces containing different cells in Tissue-in-a-Tube constructs in axial and radial configurations with clear continuity and interfaces; **b)** Failed and robust interfaces in constructs with Axial configuration; **c)** Anchor point formed with two cells in radial configuration. Addition of second bioink in the axial configuration should be done right after completion of gelation process for the first bioink. If it is added earlier, a well-defined border between two regions would not be formed, and if it is added long after this point, a firm junction would not be formed, and owing to force exerted by shrinkage of the constructs, they will tear apart. All the images in panels b and c are taken after 8 h of starting the process.

Multilayered and multimaterial tissue engineered constructs better mimic function and architecture of natural tissues [28]. Such constructs can be used to study the interaction between different cells in a tissue that happens through paracrine or contact-dependent cell signaling which significantly influences their individual function [29]. The rapid self-assembly process used in this method enables formation of constructs that can be axially or radially patterned with different cell types (demonstrated here with MCF-7 cells stained with different colors), while maintaining its structural continuity and integrity (Fig. 3a). To be able to produce axial patterns, a portion of the tubing was initially filled with the first bioink and allowed to self-assemble for 30 min which was long enough to allow the collagen to gel and solidify but not sufficient for the cells to adhere to the ECM and initiate substantial shrinkage. Next, the second bioink was introduced into the rest of the tubing which then subsequently also self-assembled and resulted in forming an axially patterned construct. The interface between the two cell regions in the tissue construct formed after shrinkage induced by the cell attachment to the ECM was found to be precise and capable of withstanding high internal tension. Delay in addition of second phase resulted in two separate unfused regions that because of high contractile forces were positioned distant from each other (Fig. 3b). Concentric or radial patterning of cells was formed by initially filling the entire space between the pins with the first bioink followed by a longer incubation time (~2 h) so that the construct shrank to nearly half of the final stable size. At this point, the excess medium was extracted and replaced with the second bioink containing a different cell type and/or ECM combination (Fig. 3a) which then proceeded to self-assemble around the partially assembled first layer in an annular fashion. Formation of second layer around the first layer at the anchor pins is shown in Fig. 3c. In radial mode, by increasing the cell number and the CMR a single layer cell coverage can be potentially made to cover the inner cell construct for example to mimic blood-brain barrier. These patterning can also be used to create constructs with different types of ECMs in different locations. While presence of collagen is necessary for formation of stable structures, other types of ECM can be mixed with it to provide more favorable environment for different cells in each region or for example to study migration of cells from one region with one type of ECM to the other. Such constructs allow direct contact between different cell types at the interface and paracrine interactions for the rest of the cells in different layers. The paracrine interactions can also be modeled in a unilateral direction by forming the constructs in two separate tubing and connecting them using interconnects. Applying a small fluid flow will allow exposure of the downstream construct to the paracrine signaling while preventing it in the upstream construct. Such cell patterning can also be useful in applications such as controlled release of pharmaceuticals [30].

**Fig. 4:**
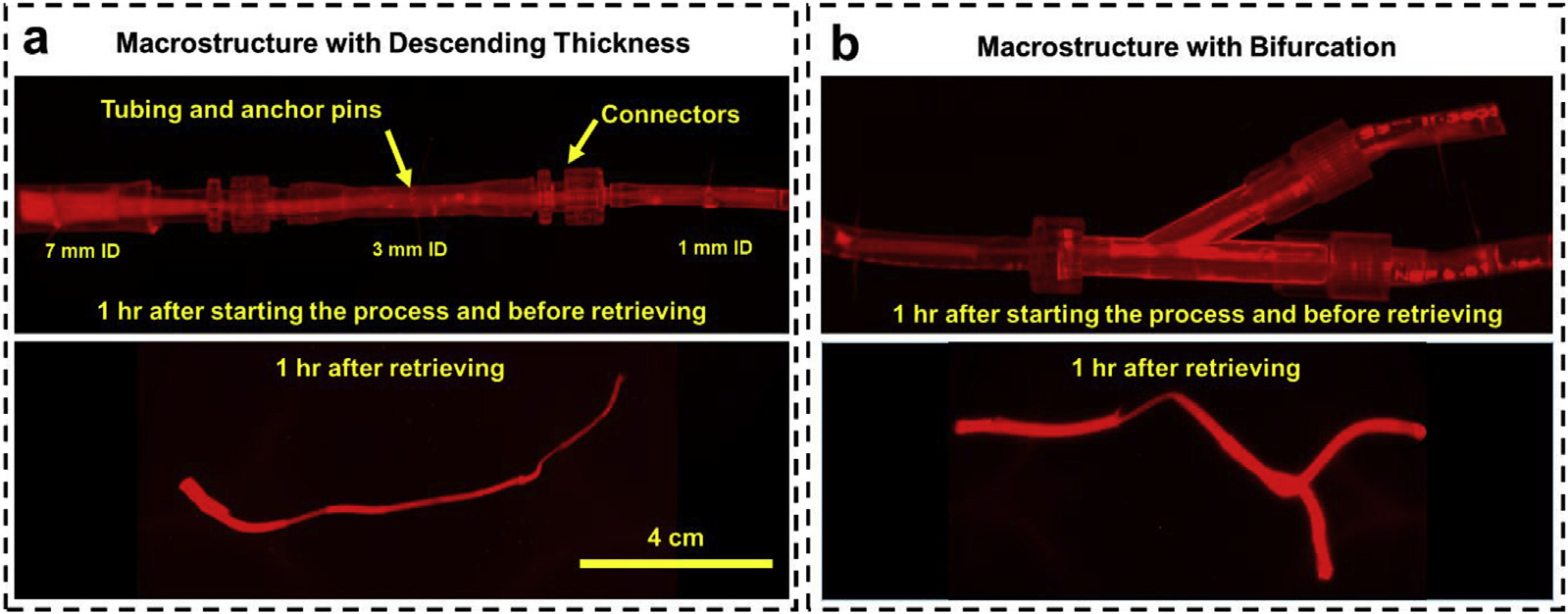
Formation of macrostructures with complex patterns using Tissue-in-a-Tube technique; **a)** long column with descending thickness and **b)** with bifurcation. Constructs are formed with HUVECs (with density of 2 × 10^6^ cells/mL and 1:3 CMR) and are stable outside the tubing. After retrieving from the tubing, a noticeable shrinkage happens but they will preserve their premeditated morphology. HUVEC, human umbilical vein endothelial cell.

Spherical constructs have been widely used, for example in the case of spheroids, mostly because of ease of fabrication for applications such as modeling the initial avascular state of cancerous tissues [7], but this format can lead to formation of necrotic core that is not favorable for other applications including modeling physiological conditions of different tissues. An alternative and elegant tissue structure would be cylindrical or tubular structures that can be extended along their axial dimension to have a larger volume without increase in the radial direction to avoid formation of necrotic cores. Control over the radial dimensions of such structures affect the mass transport fluxes within the construct and could be used to create unique biochemical environments. Here, such long tubular macrostructures with different thicknesses in different regions were fabricated (Fig. 4a) by connecting silicone tubing with different IDs, using appropriate connectors, inserting anchor pins in each of them, and then filling the entire construct with the bioink. In under an hour, the cells and ECM rapidly assembled to form constructs that are several centimeters in length but have different diameters in the various sections. The constructs were robust enough that they can be retrieved from the tubing using tweezers and were strong enough to support their own weight. These types of constructs provide different mass transfer conditions in different sections and can be used as *in vitro* models or as cell delivery vehicles for *in vivo* implantation. Extrusion printing of bioinks containing hydrogels and cells can be used to form long tubular constructs but they typically have low cell density (less than a few million cells/mL [[31], [32], [33]]). Newer extrusion techniques with lower speed can produce high-density constructs with radial and axial patterning [34,35] but have difficulty in creating branching networks and require specialized equipment. Alternatively, the hanging drop method has been modified with patterned substrates in rectangularly designed hydrophilic regions to confine cells in a semi-cylindrical fashion to assemble ECM-free fibers [36]. This interesting approach is an advancement over the traditional hanging drop method but is still limited in its ability to form multicellular patterns radially or axially. Furthermore, it requires specialized substrates and long assembly times. Our approach presented here represents a simple yet robust method that can be adapted easily to produce macro tissues of almost any length from multiple cell types with the ability to create branching structures (Fig. 4b) very easily (single step) without the use of expensive equipment or complicated operations.

The branching structures shown here (Fig. 4b) are particularly important as complex interactions between different tissues can be simulated by fabricating each tissue separately in a tubing and then simply connecting them using appropriate Y-shaped connectors. These conformations allow more complex interactions where the paracrine signaling of two different tissues can be simultaneously exposed to a common downstream tissue or inversely the signaling from a common upstream tissue could provide exposure to several downstream tissues while they do not affect each other. By repeating these connections, a more complex fluidic network can be developed that can provide physiologically relevant paracrine interactions between multiple tissue types in a simple and robust way without the use of any complex microfabrication processes. By using different cell densities in each tubing in the branched network or connecting different number of tubing containing each cell type a more accurate model of interaction between different tissues and organs can be created using proper allosteric scaling [37,38].

### 3.3 Dynamic environment

In addition to 3D cell-cell and cell-ECM interactions and paracrine activities, biophysical signals such as mechanical or electrical stimulation play an important role in recreating the *in vivo*-like microenvironments that determine the functioning of tissues [39,40]. The use of metal pins as anchors provided the ability to apply electrical stimulus to the tissue construct during different assembly and development phases. In addition, owing to the self-assembly and the contraction of the forming tissue construct that are constrained by the rigid pins, a time varying and auto-regulating mechanical stimulus is also applied on the construct. Similarly, the flexibility of the silicone tubing as well as the ability to perfuse the annular region between the tube and the tissue provided the ability to introduce active and dynamic mechanical stimulus and perfusion of fluids. A bioreactor (Supplementary Fig. 3) was designed to apply electric field (up to 5 V/cm with 50 Hz frequency) to the constructs through the anchor pins and to perfuse the growth medium to avoid waste accumulation and apply shear force. Using a microcontroller and additional pins in different locations, a range of different electrical signals can be applied at different locations, and multiple assays can be conducted while continuity of the tissue construct, and its exposure to nutrients and drugs are preserved.

**Fig. 5:**
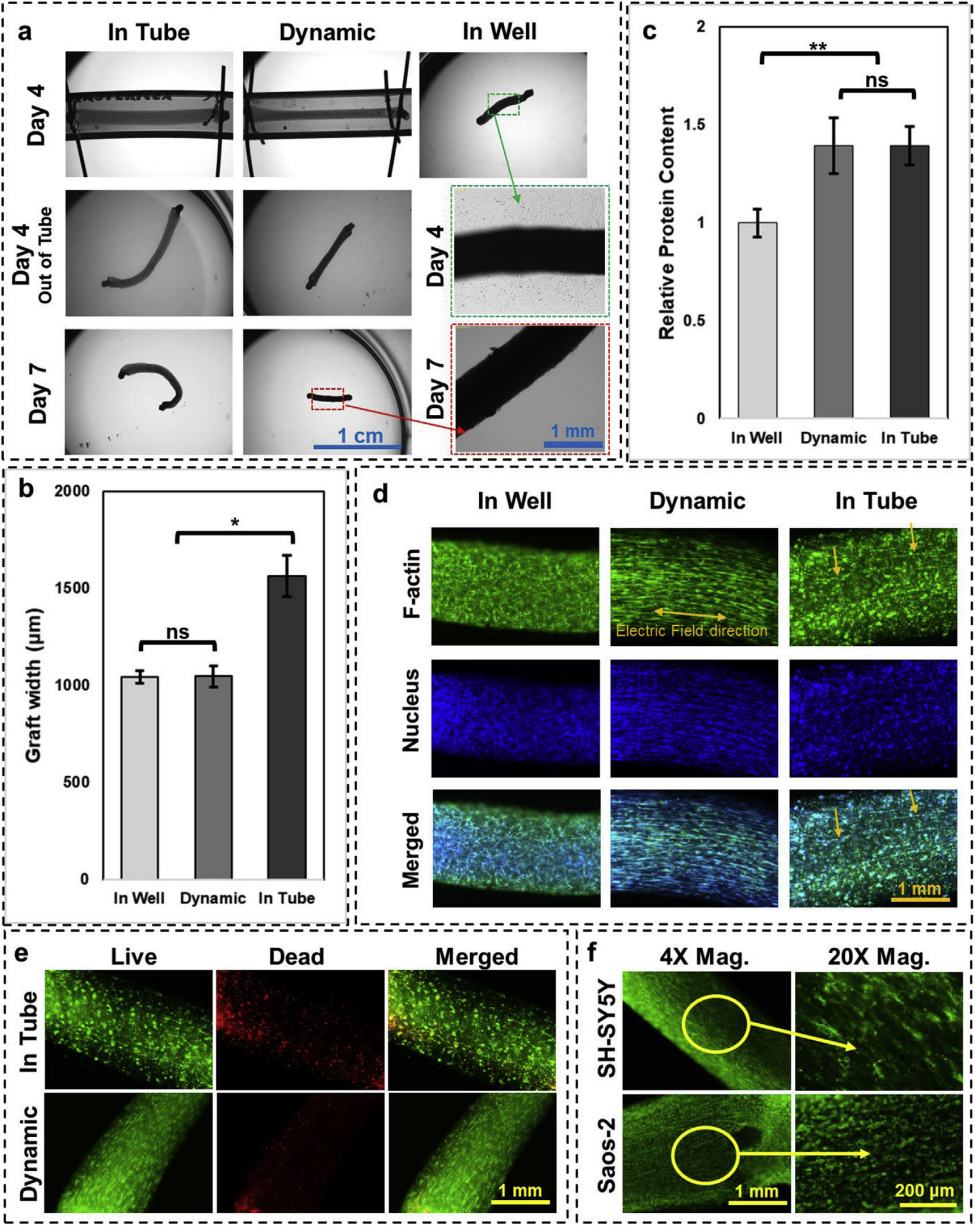
Effect of dynamic microenvironment on cellular constructs. **a)** Constructs were formed with undifferentiated myoblast C2C12s and differentiation was performed in three different conditions: ‘In Well’ with no constrictions, ‘In Tube’, constricted between anchor points, and in ‘Dynamic’ condition anchored to the pins and facing electrical stimuli; **b)** effect of culture condition on thickness of the constructs, ∗*p*-value < 0.01 (n = 4); **c)** total protein content of differentiated C2C12s in three culture conditions, ∗∗*p*-value < 0.001 (n = 4); **d)** effect of culture condition on formation of multinucleated muscle fibers and their alignment. Electrical stimulation while greatly affected the cell alignment and fiber formation, did not influence the protein content of samples. Constructs kept shrinking over time outside the constriction of tubing and its anchors, cells did not show fusion, and some of the cells even escaped the construct on to the culture plate. Samples exposed only to the anchor pins showed some fiber formation and did not show much shrinkage and cell escape after retrieving from the tubing. Samples in ‘Dynamic’ environment showed full fiber formation, had more shrinkage after retrieving from the tubing, and no cell break out was observed. **e)** Live/dead stained images of ‘In Tube’ and ‘Dynamic’ samples at day 4 right after retrieving from the tubing. There are slightly more dead cells in the ‘In Tube’ group. **f)** Effect of electric field on alignment of SH-SY5Y and Saos-2 cells.

Importance of dynamic environment on cell function was studied by studying effect of electric field on differentiation and maturation of myoblast cells as well as their ECM deposition. For this purpose, muscle tissue constructs (formed using C2C12 cells, 1:3 CMR, 2 × 10^6^ cells/mL, and 2 cm apart pins) that were formed in their growth medium and subsequently their differentiation into mature skeletal muscle cells in the form of multinucleated myofibers, in three different conditions were compared. Cellular behavior in samples formed in the tubular constructs without subsequent confinement to the anchor pins was studied by transferring the formed tubular constructs to 6 well plates containing differentiation medium (‘In Well’ group), 24 h after the process started. Effect of being confined to anchor pins on cell behavior was studied by keeping the formed tissue samples in the tubing (‘In Tube’ group) and switching to differentiation medium. Effect of electrical stimulation on this process was studied by applying electric field to the anchored samples in the tubing while they were exposed to differentiation medium (‘Dynamic’ group). Grafts in these conditions were compared 3 days later (4 days in culture in total) (Fig. 5). Bright field images of samples at day 4 (Fig. 5a) showed that ‘In Tube’ group samples had significantly higher thicknesses (1563 ± 105 μm) compared with ‘Dynamic’ and ‘In Well’ samples which were not significantly different from each other (1047 ± 55 and 1042 ± 31 μm respectively) (Fig. 5b). Measurement of total protein content of the constructs using Pierce BCA assay showed that both ‘Dynamic’ and ‘In Tube’ samples were similar to each other in protein content (Fig. 5c) which was significantly higher as compared with ‘In Well’ samples (~1.4 times higher). At day 4, ‘In Tube’ and ‘Dynamic’ samples were retrieved from the tubing and were kept in 6 well plate in differentiation medium for 3 more days. Immediately after retrieval, ‘Dynamic’ samples showed a detectable shrinkage, whereas it was much lower for ‘In Tube’ ones. After 3 more days in culture, more shrinkage was observed for ‘Dynamic’ samples, whereas ‘In Tube’ ones showed a small amount of shrinkage. Higher magnification imaging during the first 3 days of differentiation showed that a high number of cells disaggregated from ‘In Well’ group and proliferated on the well surface (Fig. 5a), whereas such disaggregation was not seen in the case of ‘In Tube’ and ‘Dynamic’ constructs during the 3 days of culture after being retrieved from tubing. Staining for F-actin in the constructs using phalloidin at day 4 (Fig. 5d) revealed that although samples in all three groups were treated with the same differentiation medium, cells in the ‘In Well’ group did not fuse and did not form multinucleated fibers unlike samples in the ‘Dynamic’ group that showed formation of fibers aligned in the direction of electric field (perpendicular to the anchor pins). Samples in the ‘In Tube’ group, which were exposed to mechanical constriction, had a few fibers formed which were very short compared with the ones in the ‘Dynamic’ group. Presence of electrical stimulation in ‘Dynamic’ group did not affect the protein content and therefore ECM production in those samples as compared with the ‘In Tube’ group where there was no electrical stimulation (Fig. 5c), but it did induce more extensive fiber formation and maturation of skeletal muscle cells. This shows that various stimulation that are important to obtain morphological features seen in natural tissues can be induced in this method easily. Presence of anchor pins provided a continuous mechanical strain to the developing construct which stabilized the construct and prevented cells from disaggregating. Live/dead staining of the ‘In Tube’ and ‘Dynamic’ samples right after retrieval from the tubing at day 4 showed fewer dead cells in the ‘Dynamic’ condition (Fig. 5e) which shows not only the ‘Dynamic’ environment promoted the differentiation and maturation of cells, it also preserved their viability as well. Although electrical stimulation and perfusion have been applied previously to skeletal muscle cells to study myofiber formation [[41], [42], [43], [44]], our method allows simultaneous application of all three types of stimuli-perfusion, electrical, and mechanical along with control over tissue interfaces, environmental factors such as construct size and compactness, as well as a fast process with little to no effect on cell viability.

Other tissue constructs including neural (formed using SH-SY5Y neuroblastoma cells) and osseous (formed from Saos-2 osteosarcoma cells) also demonstrated cellular alignment with the electrical stimulus in our tissue formation method (Fig. 5f). These constructs were kept in culture for 8 days and were able to maintain their integrity despite observation of further shrinkage. Although the effect of electric field on bone cells have been previously studied in 2D cultures [45], cells cultured on scaffolds, or substrates [46,47], here we have shown *in situ* cellular alignment of osteoblast-like cells in a truly 3D culture system composed only of cells and ECM. Such alignment can potentially be used to mimic the anisotropic microstructure of bone [48] that influences its behavior resulting in anisotropic viscoelastic properties [49]. Similarly, alignment of neuronal cells along with electric field lines have been demonstrated previously in 2D culture systems or on scaffolds [50,51] as well as in loosely packed hydrogel-based constructs [52,53]. However, our method demonstrates the ability to create highly dense and aligned neuronal tissue constructs without prefabricated scaffolds which can be used to form neural tube bundles for the use in regenerative medicine applications.

In addition to electrical stimulation, additional biophysical stimulation can also be applied in this system. For instance, Supplementary Video 1 shows other types of stimuli including the perfusion of medium that generates shear force of the fluid flow on the cells on the outer layer of the tubular construct and the mechanical bending of the tubing that translates to the mechanical deformation of the constructs including stretching or compression. As shown in the video, wave-like mechanical deformation can be created in the tissue graft by controlling the flow rate of the medium as well. Effect of mechanical deformation on maturation of skeletal muscle cells was studied by forming the C2C12 constructs and creating the dynamic environment by applying mechanical stimulation by bending to the tubing as shown in Supplementary Video 1 for 2 h every day for 3 days. Mechanical stimulation was started 1 day after grafts were formed and transferred to differentiation medium. More fibers were observed in those constructs exposed to mechanical stimulation (Supplementary Fig. 4) as compared with those without the stimulation. However, fewer fibers were formed under this mechanical stimulation conditions compared with electrical stimulation (Fig. 5d), which could be because of the shorter (only 2 h of mechanical stimulation was done every day) duration of the stimulation compared with the electrical one (applied continuously). Details of the mechanical deformation are included in the Supplementary information. Similar effect of mechanical deformation on cellular alignment and fiber formation of skeletal muscle cells and their maturation in 3D culture systems have been previously observed [43,54], but independent of stimulation mode (chemical, mechanical, or electrical) or ECM type, it has been shown that once such mature skeletal muscle cell constructs with dense and highly organized fibers are formed, the constructs can be actuated and will exert forces that can be used for applications such as soft biorobotics and bioactuators [[55], [56], [57], [58]].

Supplementary video related to this article can be found at https://doi.org/10.1016/j.mtbio.2020.100070.

The following is/are the supplementary data related to this article:

Video 1 Video 1

This technique is also compatible with high throughput screening applications. For example, a large number of constructs can be formed in the same tubing by inserting more than just two anchor pins or by connecting different construct containing tubing to each other in series. This could increase the nutrient consumption and by-product accumulation rate and adjustments to the flow rate of the medium or size of the tubing needs to be done to properly support cellular behavior. Alternatively, connections in parallel can be also used in case perfusion is not desired. This will isolate the metabolic impact of one tissue type on the other. A combination of series and parallel connections can be introduced to replicate the ratio of metabolic outputs of different tissue types in the body.

## 4 Conclusion

A new and simple biofabrication technique for rapid formation of collagenous, tubular, and macroscale tissue constructs has been developed. The method allows for formation of complex tubular shapes and branching networks while providing the flexibility to control positioning of different cell types in predefined patterns, at high densities and with clear interfaces that can mimic *in vivo–*like environments. The fabrication process is low cost, simple, and easy to adapt to create various tissue geometries and allosteric scaling. It can also be used to apply various biophysical stimuli such as mechanical deformation, fluid shear, and electric field separately or in conjunction to create a dynamic environment as well. We have demonstrated that a variety of cell types including endothelial, epithelial, skeletal muscle cells, bone cells, and neuronal cells are amenable to this method, and multicellular structures can be created by radial or axial patterning. To demonstrate the efficacy of this method, aligned muscle, neural, and bone tissues were constructed. Macrostructures (several centimeters in length) with complex patterns such as the columns with different thicknesses in different regions and bifurcated constructs which can be used as cellular constructs or *in vitro* models were rapidly constructed. By providing both the biochemical and the biophysical environment and the ability to direct complex paracrine interactions between different segments using fluid flow, these systems can serve as a versatile tool for biomedical researchers trying to understand disease mechanisms and discover new drugs.

## Data availability

The data that support the findings of this study are available from the corresponding author upon reasonable request.

## Declaration of competing interest

The authors declare that they have no known competing financial interests or personal relationships that could have appeared to influence the work reported in this article.
