# Supplementary material for: Tissue-in-a-Tube: three-dimensional in vitro tissue constructs with integrated multimodal environmental stimulation
Source: Mater Today Bio. 2020 Jul 28;7:100070. doi: 10.1016/j.mtbio.2020.100070 (PMC7452320; doi:10.1016/j.mtbio.2020.100070)
Supplement: Supplementary file 2 — Multimedia component 2 [file mmc2.pdf]

Tissue-in-a-Tube: Three dimensional *In vitro* Tissue Constructs with Integrated Multimodal Environmental Stimulation

Alireza Shahin-Shamsabadi [1], P. Ravi Selvaganapathy [1,2,*]

[1] School of Biomedical Engineering, McMaster University, Canada

[2] Department of Mechanical Engineering, McMaster University, Canada

[*] Corresponding Author: P. Ravi Selvaganapathy, Department of Mechanical Engineering, McMaster University, Canada

Supplementary Information

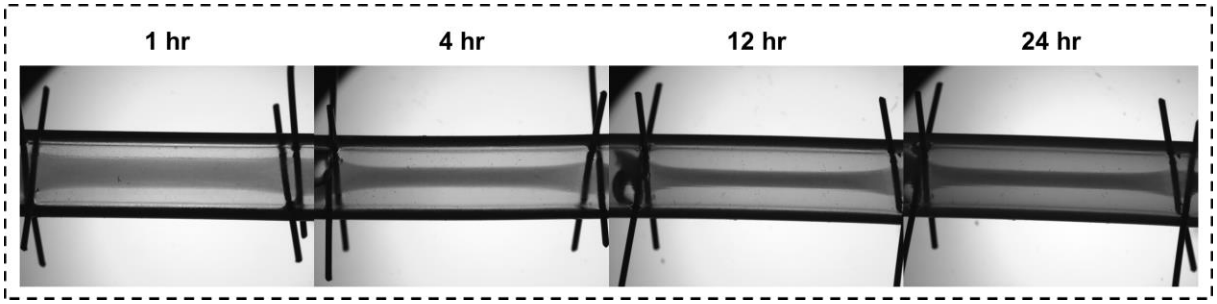

Supplementary Fig. 1. Shrinkage pattern of the constructs over time for samples formed with MCF-7 cells with 2×10^6 cells/mL and 1:3 ratio. A dramatic decrease in size is observed in the first 4 h followed by a much lower shrinkage in the next 20 h.

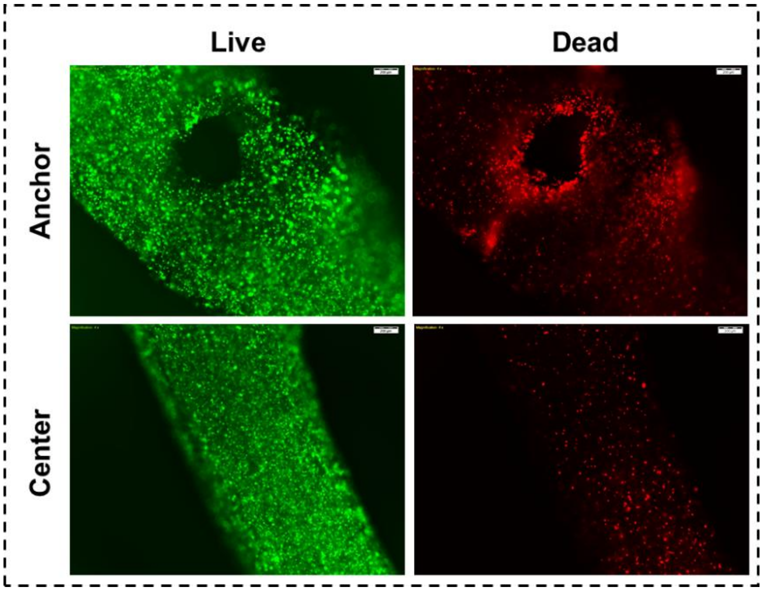

Supplementary Fig. 2. Distribution of live and dead cells in the middle of the construct vs. close to anchor points. In the locations close to the pins number of dead cells was higher while in the other regions of the construct a uniform distribution of live cells with only low number of dead cells was observed. Staining and imaging were done 4 h after the fabrication process started.

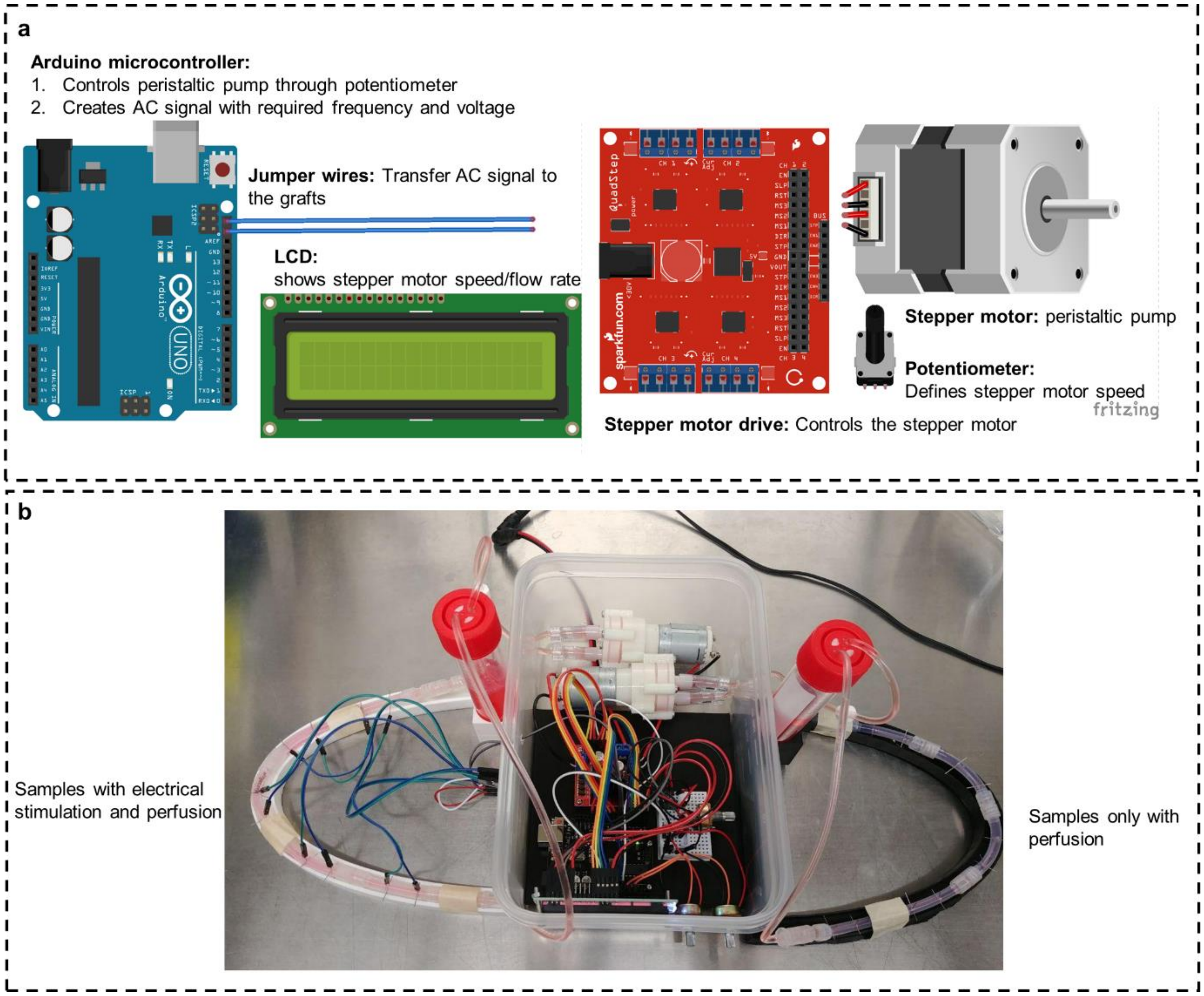

Supplementary Fig. 3. a) Components of the bioreactor. Arduino microcontroller creates the AC step signal (50 Hz and -5 to +5 V). It also controls the flow rate through the speed of the motor that can be defined using the potentiometer and is shown on the LCD. **b)** Assembled bioreactor.

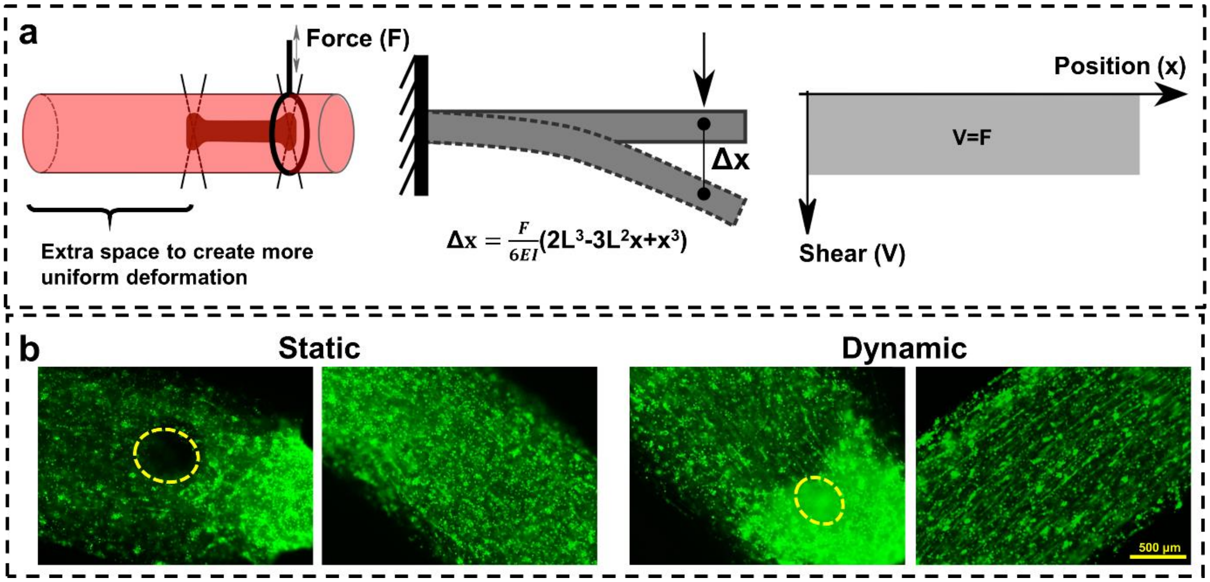

Supplementary Fig. 4. a) Schematic of the mechanical deformation imposed to C2C12 grafts to create a dynamic microenvironment, **b)** effect of mechanical deformation on fiber formation of skeletal muscle grafts in dynamic environment as compared to static condition. The tubing and graft inside it can be treated as a beam that is fixed on one side and is deflected using a concentrated force on the other end. There is a uniform shear force applied to all cross-sections of the sample across the length of the graft while there is a cubic relation between deformation and position. To create more uniform deformation in the graft, a 3 cm extra space between the left fixed side of the tubing and the graft was allowed. Formation of fibers was observed both in the middle for the sample as well as at the endpoints where connections to the pins exist. Stimulation was started one day after grafts were formed and a dynamic environment was created by deforming the tubing with amplitude of 2 cm and frequency of 0.5 Hz for 2 hr every day for three days.

Supplementary Box 1. Code used for programming the microcontroller that controls the bioreactor.

```
//define pins for the peristaltic pumps
int EnA=10; //yellow wire
int in1=9; //orange wire
int in2=8; //red wire
int EnB=11;
int in4=12;
int in3=13;
//output voltage for 1st pump
int potValue1=0;
long pwmOutput1=0;
int VoltOutput1=0;
int potValue2=0;
long pwmOutput2=0;
int VoltOutput2=0;


//using potentiometers to define speed of peristaltic pumps
int pot1=A1;
int pot2=A2;

// a step wave between pins 6 and 7 (-5 to +5V) with a frequency of 50Hz
int PinP = 6; //
int PinN = 7; //
int counter=0;


void setup() {
 //controling 1st pump
 pinMode(EnA, OUTPUT);
 pinMode(in1, OUTPUT);
 pinMode(in2, OUTPUT);
 //controling 2nd pump
 pinMode(EnB, OUTPUT);
 pinMode(in3, OUTPUT);
 pinMode(in4, OUTPUT);

 pinMode(pot1, INPUT);
 pinMode(pot2, INPUT);

 //defining outputs for the Sin wave
 pinMode(PinP1, OUTPUT);
 pinMode(PinN1, OUTPUT);
 pinMode(PinP2, OUTPUT);
 pinMode(PinN2, OUTPUT);
}

void loop() {
 //reading the potentiometer and defining the speed of 1st peristaltic pump
 potValue1 = analogRead(pot1); // Read potentiometer value
 pwmOutput1 = map(potValue1, 0, 1023, 0 , 255); // Map the potentiometer value from 0 to 255
 analogWrite(EnA, pwmOutput1); // Send PWM signal to L298N Enable pin
 digitalWrite(in1, LOW);
 digitalWrite(in2, HIGH);
```

```
 potValue2 = analogRead(pot2); // Read potentiometer value
 pwmOutput2 = map(potValue2, 0, 1023, 0 , 255); // Map the potentiometer value from 0 to 255
 analogWrite(EnB, pwmOutput2); // Send PWM signal to L298N Enable pin
 digitalWrite(in3, LOW);
 digitalWrite(in4, HIGH);

 //creating AC signal
 switcher();
}

void switcher(){
 counter=counter+1;
 if (counter%2==0){
 digitalWrite(PinP, HIGH);
 digitalWrite(PinN,LOW);
 delay (20);
 }else{
 digitalWrite(PinN, HIGH);
 digitalWrite(PinP,LOW);
 delay (20);
 }
}
```
